# Terpene Synthases in Rice Pan-Genome and Their Responses to *Chilo suppressalis* Larvae Infesting

**DOI:** 10.3389/fpls.2022.905982

**Published:** 2022-05-20

**Authors:** Yang Sun, Pei-tao Zhang, Dou-rong Kou, Yang-chun Han, Ji-chao Fang, Jiang-ping Ni, Bin Jiang, Xu Wang, Yong-jun Zhang, Wei Wang, Xiang-dong Kong

**Affiliations:** ^1^Key Laboratory for Conservation and Use of Important Biological Resources of Anhui Province, Anhui Provincial Key Laboratory of Molecular Enzymology and Mechanism of Major Diseases, College of Life Sciences, Anhui Normal University, Wuhu, China; ^2^Institute of Plant Protection, Jiangsu Academy of Agricultural Sciences, Nanjing, China; ^3^JiguangGene Biotechnology Co., Ltd., Nanjing, China; ^4^Laboratory for Biology of Plant Diseases and Insect Pests, Institute of Plant Protection, Chinese Academy of Agricultural Sciences, Beijing, China; ^5^Wuhu Qingyijiang Seed Industry Co., Ltd., Wuhu, China

**Keywords:** rice pan-genome, terpene synthase, structure variation, *Chilo suppressali*, selection pressure, differentially expressed

## Abstract

Terpene synthase (TPS) catalyzes the synthesis of terpenes and plays an important role in plant defense. This study identified 45 *OsTPS* genes (32 core genes and 13 variable genes) based on the high-quality rice gene-based pan-genome. This indicates limitations in *OsTPS* gene studies based on a single reference genome. In the present study, through collinearity between multiple rice genomes, one *OsTPS* gene absent in the reference (Nipponbare) genome was found and two *TPS* genes in the reference genome were found to have atypical structures, which would have been ignored in single genome analysis. *OsTPS* genes were divided into five groups and TPS-b was lost according to the phylogenetic tree. *OsTPSs* in TPS-c and TPS-g were all core genes indicating these two groups were stable during domestication. In addition, through the analysis of transcriptome data, some structural variations were found to affect the expression of *OsTPS* genes. Through the Ka/Ks calculation of *OsTPS* genes, we found that different *OsTPS* genes were under different selection pressure during domestication; for example, *OsTPS22* and *OsTPS29* experienced stronger positive selection than the other *OsTPS* genes. After *Chilo suppressalis* larvae infesting, 25 differentially expressed *OsTPS* genes were identified, which are involved in the diterpene phytoalexins precursors biosynthesis and ent-kaurene biosynthesis pathways. Overall, the present study conducted a bioinformatics analysis of *OsTPS* genes using a high-quality rice pan-genome, which provided a basis for further study of *OsTPS* genes.

## Introduction

Terpenes are important secondary metabolites in plants that play an important role in plant defense. Terpenes are synthesized in response to herbivore-induced (or mechanical) damage, and act as information chemicals in plant-insect interactions, thus playing an important role in plant defense. Caryophyllene is a sesquiterpene produced in response to herbivory by western corn rootworms in maize, and attracts insect-killing, predatory nematodes (Hiltpold et al., 2009). β-Ocimene is a monoterpene and an odor producing compound, that responds to herbivore-induced damage. In *Torenia*, β-ocimene synergizes with herbivory-induced plant volatiles (HIPVs) and attracts predatory mites as well as defensive insects (Shimoda et al., 2012; Valea et al., 2021). Apart from *TPS* genes, genes belong to proteinase inhibitor, lectin, chitinase, plant hormone signal transduction pathways such as JA, SA, and MAPK are also involved in plant defense against herbivores (Wang et al., 2018).

The synthesis of terpenoids is mainly catalyzed by terpene synthase (TPS), and the *TPS* is a medium-sized gene family. Except for *Physcomitrella patens*, which contains only one functional *TPS* gene, other plants contain approximately 20–152 *TPS* genes; however, some of these genes have lost their function during evolution. According to the differences in the amino acid sequences of TPSs, they can be divided into seven subfamilies, namely TPS-a, TPS-b, TPS-c, TPS-d, TPS-e/f, TPS-g, and TPS-h (Chen et al., 2011). TPS-a mainly synthesizes sesquiterpenes in monocots and dicots. For example, AtTPS21, a TPS in the TPS-a subfamily, is mainly responsible for the synthesis of (E)-β-caryophyllene in *Arabidopsis* (Abel et al., 2016). The TPSs in the TPS-b subfamily mainly synthesize monoterpenes in angiosperms. AtTPS10 yields an active monoterpene synthase enzyme, which converts geranyl diphosphate (C10) into (E)-β-ocimene, acyclic monoterpenes β-myrcene, and small amounts of cyclic monoterpenes in *Escherichia coli* (Bohlmann et al., 2000). However, almost all the genes coding the TPS-b subfamily proteins have been found in dicotyledonous plants (except sorghum) so far. TPS-c is considered to be an ancestral clade, and includes the *CPS* genes in gymnosperms and angiosperms. TPS-f is derived from TPS-e, and therefore, TPS-e and TPS-f are often called TPS-e/f subfamilies. AtTPS4 is responsible for synthesizing geranyllinalool and catalyzing the first step in the formation of the insect-induced volatile C16-Homoterpene TMTT in Arabidopsis (Herde et al., 2008). PHS1, which belongs to the TPS-e/f subfamily, was reported to mainly produce β-phellandrene and a few other monoterpenes in the tomato (Schilmiller et al., 2009). TPS-g is closely related to TPS-b, and is involved in the synthesis of acyclic monoterpenes, sesquiterpenes, and diterpenes. Three cDNAs encoding myrcene synthase and (E)-ocimene synthase (catalytically active, yielding monoterpene products) were isolated from a snapdragon petal-specific library (Dudareva et al., 2003). TPS-h only exists in non-seed plants, synthesizes diterpenoids with dual functions, and has not been found in angiosperms until now (Chen et al., 2011; Alicandri et al., 2020).

TPSs have been identified in many species. Aubourg et al. (2002) identified 40 TPSs in *Arabidopsis*, 69 TPSs were identified in grapevines (*Vitis vinifera*) through hidden Markov model (HMM) search (Martin et al., 2010), and Falara et al. (2011) identified 44 TPSs in tomato. Rice is an important food crop worldwide, as well as an important model species in plant research. Although there have been some functional studies on *OsTPSs*, they are all based on the traditional reference genomes, and some atypical TPSs that do not contain both terpene synthase N terminal and terpene synthase C terminal conserved domains, and may play an important role in certain physiological processes, are often ignored in functional studies. For example, CmMYB012, an atypical gene without SG7 or SG7-2 conserved sequences, inhibits the biosynthesis of flavones and anthocyanins in response to high temperatures in chrysanthemum, and was identified not by traditional gene family identification based on a reference genome, but by yeast one-hybrid screening (Zhou et al., 2021). The pan-genome is a consolidated database of genes in all varieties of a species. It not only contains the genes of the reference (MSU) but also that of the genes in other varieties. In addition, the pan-genome accounts for abundant structural variations (SVs). Qin et al. (2021) constructed a pan-genome of rice which contains 33 high-quality rice genomes, displays abundant presence-absence variation (PAVs), SVs, and copy number variations (CNVs), and is therefore a rich resource for studying a specific gene family. In addition, this rice pan-genome containing 33 high-quality *de novo* assembly genomes, combined with the collinear block information of genes in different varieties, can effectively identify atypical genes in a given gene family. In this study, we identified 45 TPSs in rice based on a high-quality pan-genome constructed by Qin et al. including 32 core genes and 13 variable genes. Owing to the abundant structural variations in the TPS gene family, atypical genes were identified in each rice accession. We analyzed the selection pressure on each *TPS* gene, and the influence of SVs and CNVs on *TPS* expression levels, gene structure, and conserved domains, enabling a more comprehensive study of the TPS gene family. Through the experiment of rice leaf and leaf sheath feeding on *Chilo suppressalis* larvae, 25 differentially expressed *OsTPS* genes were identified. These *OsTPS* genes affected the production of multiple intermediates, which may affect the final product of many terpene compounds. These results enable further research on *OsTPSs*, and provide a valuable resource for functional studies on TPS, as well as new insights for identifying atypical members of the gene family.

## Materials and Methods

### Rice Breeding and Treated With *Chilo suppressalis*

The rice (Nipponbare) and original population of *C. suppressalis* eggs were provided by Institute of Plant Protection, Jiangsu Academy of Agricultural Sciences (Luo et al., 2016). *C. suppressalis* were grown in the artificial climate box at 28 ± 2°C, with light-dark cycles of 16:8 h and relative humidity of 80%. An artificial diet for *C. suppressalis* larval breeding made in our laboratory using was according to Han et al. (2012) and Luo et al. (2016). The medium was cut into small pieces of 1 cm^3^, and placed into six-well plates. Neonate larvae that hatched within 2 h were transferred into six-well plates with a brush, with one larva per well. Paper towels were placed between the plate and lid to prevent the larvae from escaping.

The rice seedlings were grown individually in a cylindrical pot (25 cm and a height of 30 cm) in a greenhouse with temperature ranging from 24° to 35°C, 16 h of light/8 h of dark, and 60% humidity. Three treatments (T1, T2, and T3) were set to study the responses of rice under *C. suppressalis* infesting. T1 means that wide-type plants were induced by ten 3rd *C. suppressalis* larvae for a short time (about 5 min approximately equal to 0 h) with larvae remaining on rice. T2 means that wide-type plants were fed on by ten 3rd *C. suppressalis* larvae for 24 h with larvae remaining fed on rice. T3 means that wide-type plants were infested by ten 3rd *C. suppressalis* larvae for 24 h and cleared the trace and body of *C. suppressalis*. These treatments were performed simultaneously in the leaves and leaf sheaths of rice.

### Sample Preparation for RNA Sequencing

Total RNA was extracted from *C. suppressalis* infesting rice with the Total RNA Isolation kit (Promega, Madison, WI, United States) following the manufacturer’s instructions. Magnetic beads with Oligo (dT) were used to enrich mRNAs to construct the sequencing library. PCR amplification was performed and the AMPure XP beads were used to purify the PCR product to obtain the final library. The 24 libraries were sequenced on the Illumina NovaSeq platform.

### Identification of the Terpene Synthase Gene Family in the Rice Pan-Genome

The rice pan-genome data and the HMM file of the terpene synthase N terminal domain (PF01397) and terpene synthase C terminal domain (PF03936), were downloaded from the Rice Resource Center^1^ and Pfam protein family database,^2^ respectively. To identify the putative rice TPSs, TPS N, and C terminal domains were searched in the rice pangenome protein database (Qin et al., 2021) through HMMER 3.3.2 software, using default parameters. Putative TPS sequences were submitted to SMART,^3^ to confirm the existence of the TPS N and C terminal domains. For one gene containing TPS N and C terminal domains, the other genes in the same collinear block (syntenic genes defined by MCscan; Qin et al., 2021) were also assumed to be TPS.

### Phylogenetic Analysis

TPS sequences of *Arabidopsis* were downloaded from TAIR.^4^ A total of 44 TPS protein sequences from MSU and 1 TPS from 02428 were aligned with AtTPS by MAFFT v7.490 (Katoh and Standley, 2013), using default parameters. The phylogenetic tree was constructed using the maximum likelihood (ML) method and the Jones–Taylor–Thornton (JTT) model, using FastTree 2.1.11 software (Price et al., 2009). The final phylogenetic tree was processed using Adobe Illustrator software.

### Terpene Synthase Presence-Absence Variation Analysis

The data on the presence and absence of *TPS* genes were obtained from the study by Qin et al. (2021). The *TPS*s present in all accessions were defined as core genes. In contrast, *TPS*s present in more than 1 but less than 33 accessions were defined as variable genes.

### Evolutionary Analysis of *Terpene Synthase* Genes in 33 Rice Assemblies

The protein and CDS sequences files of the 33 rice assemblies were downloaded from the Rice Resource Center (see text footnote 1). The Ka/Ks ratio of each TPS in the 33 assemblies was calculated using the KaKs calculator 2.0 version.^5^ Ridgeline plot was drawn using the ggridges and ggplot packages in R (4.0.3 version). Heatmap was drawn based on the ratio of the number of accessions in gene pairs with Ka/Ks values greater than 1, to the total number of gene pairs.

### Information Display of Structural Variations and Copy Number Variations

Information on TPSs influenced by SVs and CNVs was obtained from the Rice Resource Center (see text footnote 1). Based on SV insertion and deletion information and CNV information, we observed the variation in TPS in different varieties using Microsoft Office PowerPoint. Gene expression data was obtained from the study by Qin et al. (2021). Genes with expression levels significantly altered between TPSs with SVs and TPSs without SVs in roots and shoots, were used to prepare column graphs using the GraphPad Prism software version 8.0.2. Statistical significance was set at ^**^p < 0.01 or ^***^p < 0.001. The proportion of SVs and CNVs associated with altered expression of TPS (*p* < 0.05 and | *r*| > 0.5) was used to draw a pie figures.

### Terpene Synthase Sequence Analysis Based on Structural Variations and Copy Number Variations

Based on TPSs which overlapped with SVs, we performed gene structure analysis in different varieties using TBtools (version 1.09) (Chen C. et al., 2018). The motifs of TPS sequences were identified using the MEME suite.^6^ The number of motifs was set to 10.

### Transcriptome Analysis

The sequencing adaptors and low-quality sequences were removed by fastp (Chen S. et al., 2018). The clean data were mapped to the nippobare genome using HiSAT2 (Kim et al., 2019). The DESeq2 (Love et al., 2014) was used for differential expression analysis, and | log_2_ ratio| ≥ 1 and FDR < 0.05 were used as a cut-off for significant differential expression. Expression correlations between transcription factor (TFs) and *OsTPS* genes were analyzed by Pearson’s correlation test (FDR < 0.05).

## Results

### Identification and Phylogenetic Analysis of Terpene Synthase in *Oryza sativa* Gene-Based Pan-Genome

Using the high-quality gene-based rice pan-genome constructed by Qin et al. (2021), we searched for the TPS N terminal domain (PF01397) and TPS C terminal domain (PF03936) in using HMM search. The protein sequences of TPS candidates were submitted to SMART (see text footnote 3) to confirm the existence of two TPS conserved domains, and 45 TPSs were identified in the rice pan-genome (Supplementary Table 1).

PAV, a type of genetic variation among different varieties of rice, is one of the genetic factors that determine rice agronomic traits. Using PAV, we can identify the absent genes in the reference genome when performing an analysis based on the pan-genome. A total of 44 TPS were identified in the reference genome, while 1 was found to be absent, using MSU as the reference genome. According to the protocol for defining core genes and variable genes used by Qin et al. (2021), 32 TPSs were identified as core genes, while 13 were identified as variable genes.

**FIGURE 1:**
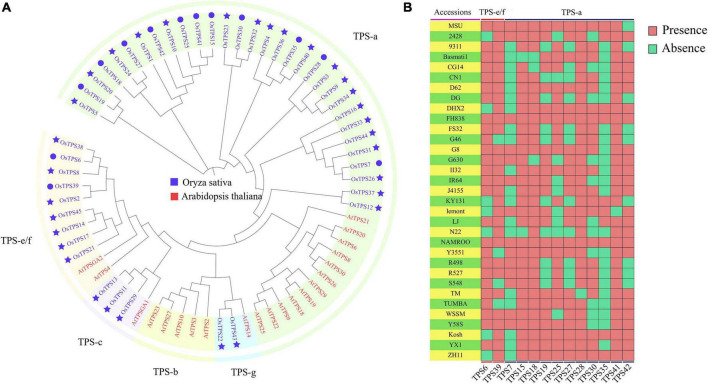
**(A)** phylogenetic tree of TPSs from *Arabidopsis* and rice. OsTPSs are marked in blue and AtTPSs are marked in red. The circles and stars in front of the OsTPSs indicate variable genes (present in more than 1 but less than 33 accessions) and core genes (present in all accessions), respectively. **(B)** Heatmap showing variable *TPS* genes present or absent in the 33 accessions.

A phylogenetic tree of TPSs from *Arabidopsis* and rice was constructed. Based on AtTPSs (Aubourg et al., 2002), OsTPSs were divided into four groups (TPS-a, TPS-c, TPS-g, and TPS-e/f). The TPS-b in *Arabidopsis* was lost in rice in the course of evolution. We found that all the TPSs from the TPS-c and TPS-g subfamilies were core genes, indicating that TPS-c and TPS-g were more stable during the process of domestication (Figure 1A). Figure 1B shows the PAVs of 13 variable TPSs, indicating that the presence and absence of TPSs in each accession was different. FH838 and NAMROO contained all TPSs. However, N22 lost eight TPSs, which was the highest loss s of TPSs among the 33 accessions. TPS41, the most stable gene among all the variable TPSs, was absent only in Lemont. In contrast, TPS35, which was absent in 23 accessions, had the highest absence variation among all the variable TPS genes (Figure 1B).

### Terpene Synthase Selection Pressure Analysis

**FIGURE 2:**
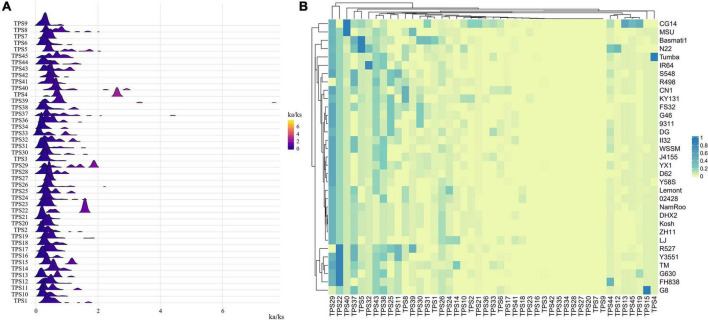
The evolution of TPS in the 33 rice varieties. **(A)** Ridgeline plot of Ka/Ks ratios of each TPS in the 33 accessions. **(B)** Heatmap of the frequency of occurrence of different rice varieties at each TPS with Ka/Ks ratio > 1.

To analyze the selection pressure of OsTPSs during evolution, we calculated the non-synonymous to synonymous substitution (Ka/Ks) ratios of each OsTPS in the 33 accessions (Figure 2). The Ka/Ks ratios of most TPS genes were <1 (Figure 2A). Furthermore, there were a few TPS genes (such as TPS33) whose Ka/Ks were < < 1, suggesting that these TPSs have important biological functions in the growth and development of rice, thereby leading to elimination of harmful mutant alleles through negative purifying selection. The peaks of the Ka/Ks ratios of TPS22 and TPS29 were located to the right of 1, indicating that these two genes were under strong positive selection pressure during the process of differentiation of the 33 rice varieties (Figure 2A). Interestingly, the Ka/Ks ratios of TPS4 in some rice varieties are > > 1, indicating rapid evolution of TPS4was happened in some rice accessions owing to the selection, which is worthy of further functional research. In addition, the proportion of TPS22 with the Ka/Ks ratio > 1 accounted for a large proportion in R527, Y3551, TM, G630, and FH838 genomes (Figure 2B), indicating that TPS22 is under positive selection pressure in these accessions. The number of gene pairs with Ka/Ks ratios > 1 in TPS16, TPS3, TPS 42, TPS 35, TPS 34, TPS 28, TPS 27, TPS 20, TPS 7, and TPS 9, was almost 0 (there were only two pairs in TPS3, and one pair in TPS16, whose Ka/Ks ratio was > 1). In addition, these TPS genes belong to the TPS-a subfamily, implying that the TPSs in TPS-a were under purifying selection pressure.

### The Terpene Synthase Gene Family Is Affected by Structural Variations and Copy Number Variations

**FIGURE 3:**
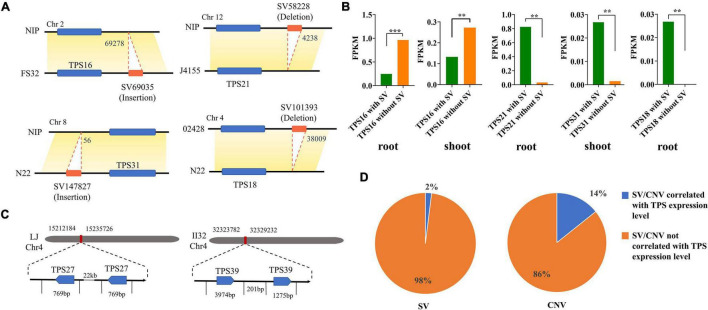
SVs and CNVs overlapping with the TPS gene family. **(A)** The display of SV insertion or deletion 2 kb upstream or downstream of *TPS* genes. **(B)** SV insertion or deletion significantly altered some of the *TPS*s expression in roots or shoots (**p < 0.01 and ***p < 0.001, student’s *t*-test). **(C)** Schematic diagram of the occurrence of CNVs that significantly altered *TPS* expression. **(D)** Pie chart showing the proportion of SVs or CNVs that significantly altered *TPS* expression levels take up total SVs or CNVs that overlapped with the TPS gene family.

Abundant SVs and CNVs were identified by aligning 32 high-quality rice genomes with reference genome (MSU) in the study by Qin et al. (2021). A total of 199 SVs were found to be related to the TPS gene family, 46, 41, 20, 22, and 70 of which overlapped with the region 2 kb upstream of the start codon, region 2 kb downstream of the stop codon, coding region, containing region, and intro sequences of *TPS*, respectively (Supplementary Table 2). SVs and CNVs affect the expression of these genes by changing the composition or position of the cis-regulatory sequences adjacent to them (Chiang et al., 2017). Qin et al. (2021) sequenced RNA from the roots and shoots of 33 accessions, and we analyzed the effects of SVs and CNVs on *TPS* expression levels based on this RNA sequencing data (Supplementary Table 4). SV69035 had a 69,278 bp insertion 2 kb upstream of *TPS16* in 19 accessions (such as FH32), causing the expression levels to significantly decrease in roots and shoots. In the region 2 kb downstream of *TPS21*, 4,238 bp (SV58228) was absent in the J4155, CN1, and Y58S genomes, which significantly decreased the expression levels. SV147827 was found to have 56 kb inserted 2 kb upstream of *TPS31* in the N22, D62, TM, and II32 genomes, which significantly increased the expression levels in shoots. A 38,009 bp deletion (SV101393) was found 2 kb downstream of *TPS18* in 02428, N22, and Lemont genomes, leading to significantly decreased *TPS18* expression in the roots. These results suggest that some SVs affected the expression levels of *TPS*s (Figures 3A,B). By counting the proportion of *TPS*s whose expression levels were significantly affected by SVs and CNVs, we found that CNVs had a greater influence on the expression of *TPS*s than SVs (Figures 3C,D).

### Structural Variations Affect the Gene Structure of Terpene Synthases

**FIGURE 4:**
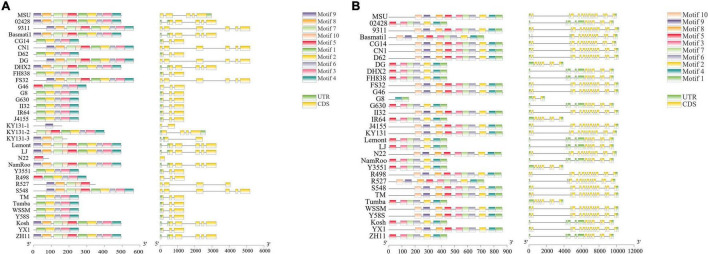
SVs affecting the gene structure of TPS9 **(A)** and TPS29 **(B)** in 33 rice accessions.

To explore whether SVs affected the gene structure in the TPS gene family, we analyzed the gene structures of *TPS*s in 33 accessions using TBtools (Supplementary Figure 1). The results showed that the gene structures in some of the *TPS*s which overlapped with the SVs were altered. For example, motif 9 in *TPS9* was absent in 18 accessions, including CG14, D62, and FH838, and there was only one motif in KY313 and N22; CDS and UTR also showed corresponding changes (Figure 4A). These results indicate that gene truncations have been a common occurrence in the evolution of *TPS9*, and are likely to lead to interesting changes in its function, such as the appearance of new functions, or pseudogenization (Flagel and Jonathan, 2009). It is worth noting that large-scale structural variations, especially large deletions of protein-coding regions, lead to loss of the typical functional domain of the gene family, causing the gene to be ignored during gene family analysis based on a specific reference genome, and thus obscuring its possible function in the phenotype. For example, *TPS9* would be ignored during gene family analysis when CG14 genome was used as the reference genome. In this study, this problem was effectively addressed by analyzing the pan-genome containing 33 high-quality genomes. The collinear block information obtained through the collinearity region between the 33 accessions, allowed us to analyze the sequence and structure of each TPS gene in each accession. In addition, *TPS29* lacked 3 motifs in 14 accessions, including 02428, DG, and so on (Figure 4B). These results indicate that SVs altered the gene structure of *TPS*s, and the common occurrence of gene truncations in the TPS gene family warrants further functional research.

### Structural Variations Caused a Large Number of Atypical Terpene Synthases in 33 Rice Accessions

**FIGURE 5:**
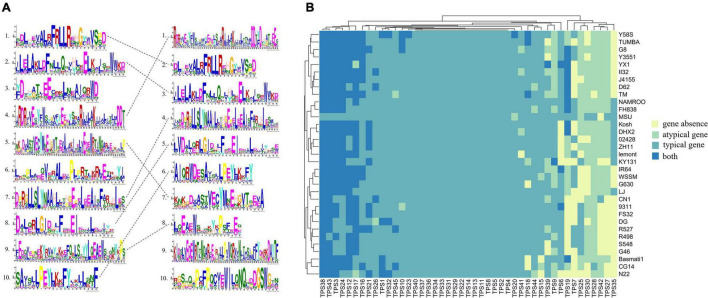
SVs affecting the conserved domains of TPSs. **(A)** The conserved domain sequence logos of TPSs from reference (left) and Basmati1 (right) genomes. The dotted line represents the correspondence between Basmati1 and the reference genome. The 10 conserved domains are arranged in ascending order of *E*-values. **(B)** The typical and atypical TPS genes in each accession. The word “both” represents that there were both typical and atypical genes in each accession.

SVs are the main factor behind the changes in the protein spatial folding structure (Al-Shatnawi et al., 2015). To explore whether SVs affected the conserved domains of TPSs, we uploaded the TPS amino acid sequences of Basmati1, which has the most TPSs overlapping with SVs, to MEME (see text footnote 6), and compared them with the conserved domains of 44 TPSs from MSU and 1 TPS from 02428 (Figure 5A). In general, two of the ten conserved domains did not correspond with each other, and the conservative order of the eight conserved domains was altered. Furthermore, the consistency of amino acid changes in each group of corresponding sequence logos of motifs, suggested that SVs had a strong influence on the conserved domains. The changes in conserved domains always hinder the identification of the members of a gene family, as they can be ignored in functional studies. Therefore, we also counted and displayed atypical genes (not containing TPS N and C terminal domains simultaneously) in each accession (Figure 5B). The results showed that TPS38, TPS43, TPS3, TPS24, TPS12, TPS17, and TPS16 in most accessions were both typical and atypical genes. TPS25, TPS30, TPS28, TPS42, and TPS27 were atypical genes in many accessions, which would be ignored in traditional gene family analysis based on single genome references. Therefore, it is necessary to perform gene family identification using gene-based pan-genomes. The above results indicated that SVs affect the conserved domains of TPS, and are thus responsible for the appearance of a large number of atypical genes in the 33 accessions.

### Responses of the *OsTPS* Genes Under the Infesting of *Chilo suppressalis* Larvae

**FIGURE 6:**
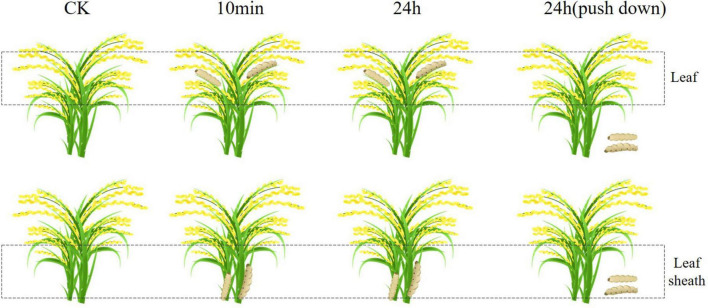
The experimental setup. Three treatments (T1, T2, and T3) were set to study the responses of rice under *C. suppressalis* infesting.

**FIGURE 7:**
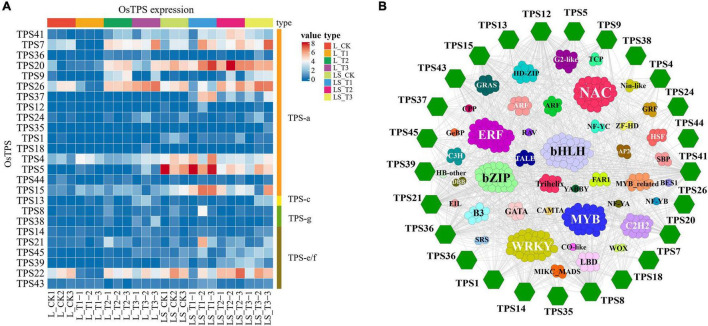
**(A)** Heatmap of the differentially expressed OsTPS genes. **(B)** Coexpression network of OsTPS genes with transcription factors. The hexagons represent the OsTPS genes. Correlations between TFs and OsTPS genes are calculated by Pearson’s correlation test (FDR < 0.05).

Through the experiment of rice leaf and leaf sheath feeding on *Chilo suppressalis* (Figure 6), the transcriptome of rice leaf and leaf sheath were sequenced. By comparing the feeding samples with the control, 25 differentially expressed *OsTPS* genes were identified (Figure 7A). The expression of OsTPS20 in the leaf sheath of *Chilo suppressalis* was higher than that of the control at three time points after feeding, indicating that it was activated by insect feeding. In the present study, the Pearson correlation coefficient between rice transcription factors and differentially expressed *OsTPS* genes was calculated. The results showed that several transcription factor members such as bHLH, WRKY, and bZIP were involved in the regulation of *OsTPS* genes (Figure 7B).

**FIGURE 8:**
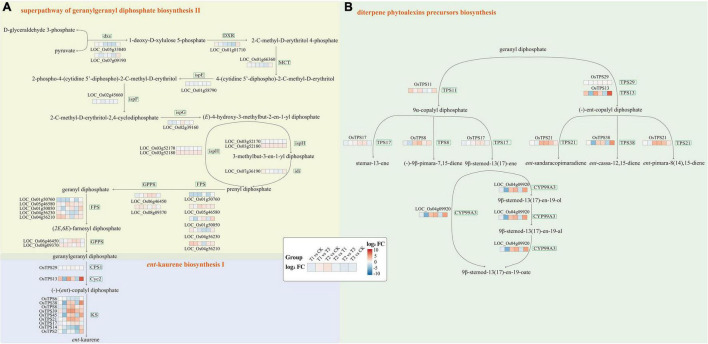
The expression of key genes in and ent-kaurene biosynthesis pathways **(A)** and diterpene phytoalexins precursors biosynthesis **(B)**.

In the diterpene phytoalexins precursors biosynthesis and ent-kaurene biosynthesis pathways, several *OsTPS* genes were differentially expressed in leaves (Figures 8A,B). In the process of ent-kaurene biosynthesis, many *OsTPS* genes were not responded at the early stage of *Chilo suppressalis* feeding, but many *OsTPS* genes such as OsTPS38 (LOC_Os02g36140), OsTPS8 (LOC_Os04g10060), OsTPS39 (LOC_Os04g52210) and OsTPS21 (LOC_Os12g30824) were up-regulated 24 h after feeding. Among them, the expression of OsTPS38 was further increased in the samples which removed the *Chilo suppressalis* after 24 h of feeding. Geranylgeranyl diphosphate was needed for ent-kaurene synthesis, and its biosynthesis-related genes were also affected by the *Chilo suppressalis* feeding. LOC_Os04g56230, an FSP (farnesyl diphosphate synthase) gene involved in the synthesis of prenyl diphosphate, had the highest expression level at the early stage of feeding. With the increase of feeding time, the expression level of Os04g56230 was still higher than the control samples, but was lower than the early stage samples. The expression pattern of another FSP gene LOC_Os01g50760 was opposite to that of Os04g56230. It indicated that gene family members didn’t play the same role in response to external stresses.

In the process of diterpene phytoalexins precursors biosynthesis, many genes encode the metabolites synthesis enzymes responded to the feeding of *Chilo suppressalis*. Such as *OsTPS21* (LOC_Os12g30824), which encode the enzyme of ent-sandaracopimaradiene and ent-pimara-8 (14), 15-diene synthesis, was up-regulated at 24 h after feeding. The (-)-ent-copalyl diphosphate synthesis related gene *OsTPS13* (LOC_Os02g36210) was also up-regulated after feeding, especially in the samples which removed the *Chilo suppressalis* after 24 h of feeding. LOC_Os04g09920, a kind of 9-beta-pimara-7,15-diene oxidase, was also highly expressed in the leaves of 24 hours *Chilo suppressalis* feeding. It affected the production of 9β-stemod-13 (17)-en-19-oate, which was involved in the diterpenoid biosynthesis.

## Discussion

Chen et al. (2011) established an HMM model of TPS, based on multiple sequence alignment of seven representative full-length TPS proteins (PpCPS/KS, PgCPS, PaTPS-Lim, PtTPSFar, At1g61680, At5g2390270, and At5g2390270), and identified 33 TPSs using hmmsearch. However, we identified 45 TPSs by hmmsearch and SMART, and confirmed TPS N and C terminal domains based on the high-quality rice gene-based pan-genome constructed by Qin et al. (2021) A total of 42 TPSs were identified in the reference genome, which was more than the number of TPSs identified by Chen et al. (2011). The reference genome used in our study was Nipponbare V.7.0 whose quality had been significantly improved compared to the reference genome used by Chen et al. (2011). Therefore, TPSs were identified more accurately in this study. Furthermore, since the gene-based pan-genome constructed by Qin et al. contains 32 additional high-quality rice genomes apart from the reference genome, we identified TPS42, which was lost in the reference genome. In addition, when identifying TPSs, homologous genes that were in the same collinear block were considered as TPS for a gene that contained both TPS N and C terminal domains. For example, TPS20 and TPS25 are atypical genes in MSU and do not contain both N and C terminal domains. Because their homologous genes are typical genes (genes with both N and C terminal domains), these two atypical genes were identified as TPSs in our study. However, these atypical genes cannot be identified when performing gene family identification using a single reference genome. SV is a common genetic variation, and the changes in conserved domains caused by SVs may lead to the omission of gene family members in the process of reference genome-based gene family identification. For example, Huang et al. found that AtVRLK1 belongs to the RLK gene family and regulates cell wall thickening, and while the phenotype was weak in single and triple knockout mutants, it was strong in the dominant-negative mutant (Huang et al., 2018), indicating that functional pathways may involve other members of the RLK gene family which due to changes in their conserved domains, cannot be identified through traditional gene family analysis based on a single reference genome. Man et al. (2020) developed an improved gene discovery method based on phylogenetic inference and iterative HMM searching, that can identify atypical genes with changes in gene structures and protein domains. However, the gene family identification without reconfirmation of conserved domains cannot guarantee the accuracy of the identified gene family members. Therefore, it is necessary to perform gene family identification using gene-based pan-genomes. The above results indicated that SVs affect the conserved domains of TPS, and are thus responsible for the appearance of a large number of atypical genes in the 33 accessions.

Generally, gene family identification requires searching for conserved domains in a genome protein file using hmmsearch, submitting the presumed gene protein to SMART to reconfirm the existence of the conserved domain, and finally obtaining the gene family members. However, some genes in a gene family do not contain conserved domains, and these genes are filtered in the process of conserved domain search and reconfirmation using hmmsearch and SMART. Nonetheless, these genes lacking conserved domains may also perform important functions. For example, Zhou et al. found an atypical gene (without SG7 and SG7-2 domains at its C-terminal) called CmMYB012, which plays an important role in response to high temperature in chrysanthemum (Zhou et al., 2021). It remains to be determined whether the reconfirmation of conserved domains improves the accuracy of gene family identification, or ignores some functional members of the gene family. In this study, we identified the TPS gene family based on a high-quality gene-based pan-genome, which not only confirmed the conserved domains of all members of the TPS gene family using hmmsearch and SMART, but also identified the atypical genes through collinear block information of 33 accessions, which can be more convenient, effective, and accurate for gene family identification and sequence enrichment. According to the TPS gene sequences with presence/absence variations, a series of experiments can be performed to study the effects of overexpression or silence of these genes on the production of terpenes in rice.

There have been many functional studies on OsTPSs. For example, OsTPS33 has been found to catalyze the synthesis of volatile sesquiterpenes, and plays an important role in the process of indirect defense in rice (Cheng et al., 2007). In this study, *TPS33* is identified as a core gene, and the Ka/Ks ratio of 33 rice accessions is < < 1, suggesting that *TPS33* is stable and may participate in basic biological processes in rice, and has been strongly purified by selection pressure during evolution. SV and CNV analyses also showed that *TPS33* neither overlaps with SV, nor does any CNV occur in it, which further indicates a high level of conservation in the gene. Zhan et al. found that OsTPS35 was positively selected in *japonica*. In the presence of Mg^2+^, OsTPS35 catalyzes the conversion of geranylgeranyl diphosphate (GGDP) to casbene. An approximately 1.9-fold increase in 5,10-diketo-casbene levels was observed upon overexpression of *OsTPS35*, while it was reduced to undetectable levels upon the knockout of *OsTPS35* (Zhan et al., 2020). Casbene is associated with rice resistance. For example, 5,10-diketo-casbene, a casbane-type diterpene phytoalexin in rice, plays an important role in resistance to bacterial blight and rice blast (Inoue et al., 2013). PAV heatmap showed that OsTPS35 was mainly absent in *indica* (Figure 1B). In addition, the region containing TPS35 overlaps with 10 SVs, and these 10 SVs were mostly deleted in *indica*. These results indicate that TPS35 and SVs overlapping with TPS35 were under different selection pressures in *japonica* and *indica*. OsTPS24 improves the resistance of bacterial leaf blight by encoding a jasmonate-responsive monoterpene synthase, which can damage the cell membrane of Xoo (Yoshitomi et al., 2016). We found that *OsTPS24* overlapped with nine SVs, and most of the overlapping regions were 2 kb upstream of the start codon. In this study, certain SVs significantly altered the expression levels of TPSs in roots and shoots. Therefore, whether or not the SVs overlapping with TPS24 affect TPS24 expression in other tissues requires further investigation. Although TPS24 is a typical gene on the reference genome, there are both typical and atypical genes in other rice varieties, such as Basmati1 and CG14. Since atypical genes may have important functions, finding whether or not these atypical genes play a role in different varieties requires further in-depth research.

Ent-kaurene is a class of diterpene metabolites, is an important intermediate in the synthesis of gibberellin (Su et al., 2017; Yang et al., 2020). Diterpenes are not only important intermediates, but also play important roles in the interaction between plants and other organisms. For example, diterpenoid kauralexins is involved in herbivore and antifungal defense (Schmelz et al., 2011). Some diterpenoids can be used as insecticides, such as Rhodojaponin III, grayanotoxin III and kalmanol, which show antifeedant, growth inhibition and insecticidal activities against *Leptinotarsa decemlineata* and *Spodoptera frugiperda* larvae (Klocke et al., 1991). As a substance that interacts with insects in plants, ent-kaurene has not been deeply studied. In the present study, several *OsTPS* genes (OsTPS38, OsTPS8, OsTPS39 etc.) that synthesize ent-kaurene were up-regulated in rice after 24 h *Chilo suppressalis* feeding. The higher expression of these genes may suggest their potential role in insect resistance. In an ecological context, some diterpenoids have been proved to have insect resistance. For example, diterpene phytoalexins have been found to have a variety of biological functions in rice and maize, including allelopathy mediated by root exudates and insect antifeedant activity (Schmelz et al., 2014). During the diterpene phytoalexins precursors biosynthesis, several differentially expressed *OsTPS* genes affected the production of multiple intermediates, which may affect the final production of diterpene phytoalexins. In another Stem Borer (SSB), Chilo suppressalis (Walker) Infestation study, many other genes, such as *LTPL164*, *LTPL151*, and *LOC Os11g32100* were showed that they have a potential role in insect resistance (Wang et al., 2018).

In conclusion, this is an in-depth study on the TPS gene family, based on a high-quality rice gene-based pan-genome, and revealed the abundant variations and possible changes in rice traits caused by these variations, providing useful resources and knowledge for rice resistance breeding.

## Data Availability Statement

The data are presented in the manuscript and the supporting materials. The raw reads data are submitted to the Short Read Archive (SRA) and BioProject accession number PRJNA815937.

## Author Contributions

YS, X-DK, WW, J-CF, and Y-JZ conceived and designed the experiments. YS, D-RK, P-TZ, and BJ contributed to manuscript writing. YS, P-TZ, J-PN, Y-CH, XW, and D-RK conducted the experiments. YS, J-PN, Y-CH, XW, Y-JZ, and X-DK contributed to the data analysis. All authors contributed to the article and approved the submitted version.

## Conflict of Interest

J-PN and X-DK were employed by the JiguangGene Biotechnology Co., Ltd. WW was employed by the Wuhu Qingyijiang Seed Industry Co., Ltd. The remaining authors declare that the research was conducted in the absence of any commercial or financial relationships that could be construed as a potential conflict of interest. The reviewer LH declared a shared affiliation with the author Y-JZ to the handling editor at the time of review.

## Publisher’s Note

All claims expressed in this article are solely those of the authors and do not necessarily represent those of their affiliated organizations, or those of the publisher, the editors and the reviewers. Any product that may be evaluated in this article, or claim that may be made by its manufacturer, is not guaranteed or endorsed by the publisher.

## Footnotes

1
https://ricerc.sicau.edu.cn/

2
http://pfam.xfam.org/

3
http://smart.embl-heidelberg.de/

4
https://www.arabidopsis.org/

5
http://services.cbu.uib.no/tools/kaks

6
http://meme-suite.org/tools/meme

## Supplementary Material

Supplementary Figure 1 Gene structure of the TPSs overlapped with SVs. **(A–H)** Were the gene structure of TPS2, TPS8, TPS11, TPS14, TPS 17, TPS21, TPS28, and TPS45 respectively.
